# The positive and negative emotion functions related to loneliness: a systematic review of behavioural and neuroimaging studies

**DOI:** 10.1093/psyrad/kkad029

**Published:** 2023-11-24

**Authors:** Qianyi Luo, Robin Shao

**Affiliations:** Department of Clinical Psychology, The Affiliated Brain Hospital of Guangzhou Medical University, Guangzhou 510370, P.R. China; Key Laboratory of Neurogenetics and Channelopathies of Guangdong Province and the Ministry of Education of China, Guangzhou Medical University, Guangzhou 511436, P.R. China; Department of Affective Disorder, The Affiliated Brain Hospital of Guangzhou Medical University, Guangzhou 511370, P.R. China; Key Laboratory of Neurogenetics and Channelopathies of Guangdong Province and the Ministry of Education of China, Guangzhou Medical University, Guangzhou 511436, P.R. China

**Keywords:** loneliness, positive emotion, negative emotion, emotion regulation, social emotion, striatum, prefrontal cortex

## Abstract

Loneliness is associated with high prevalences of major psychiatric illnesses such as major depression. However, the underlying emotional mechanisms of loneliness remained unclear. We hypothesized that loneliness originates from both decreases in positive emotional processing and increases in negative emotion processing. To test this, we conducted a systematic review of 29 previous studies (total participants *n* = 19 560, mean age = 37.16 years, female proportion = 59.7%), including 18 studies that included questionnaire measures of emotions only, and 11 studies that examined the brain correlates of emotions. The main findings were that loneliness was negatively correlated with general positive emotions and positively correlated with general negative emotions. Furthermore, limited evidence indicates loneliness exhibited negative and positive correlations with the brain positive (e.g. the striatum) and negative (e.g. insula) emotion systems, respectively, but the sign of correlation was not entirely consistent. Additionally, loneliness was associated with the structure and function of the brain emotion regulation systems, particularly the prefrontal cortex, but the direction of this relationship remained ambiguous. We concluded that the existing evidence supported a bivalence model of loneliness, but several critical gaps existed that could be addressed by future studies that include adolescent and middle-aged samples, use both questionnaire and task measures of emotions, distinguish between general emotion and social emotion as well as between positive and negative emotion regulation, and adopt a longitudinal design that allows us to ascertain the causal relationships between loneliness and emotion dysfunction. Our findings provide new insights into the underlying emotion mechanisms of loneliness that can inform interventions for lonely individuals.

## Introduction

Loneliness refers to an individual's subjective perception of unsatisfied social needs and relationships (Cacioppo *et al*., 2015). Loneliness is a prevalent condition that affects populations of all ages (Cacioppo *et al*., 2015; Loades *et al*., 2020; Van As *et al*., 2022). Substantial evidence indicates close associations between high loneliness levels and various major physical and psychological illnesses, such as dementia, cancer, cardiovascular diseases, major depression, and anxiety disorders (Hawkley and Cacioppo, 2010; Ernst *et al*. 2021; Shen *et al*., 2022; Liang *et al*., 2023). Therefore, high rates of loneliness pose significant health threats and burdens to both individuals and modern society (Holt-Lunstad *et al*., 2015). It is widely considered that loneliness originates from and exacerbates negative emotions about social relationships and interactions, which cause lonely individuals to withdraw and disconnect from social networking (Cacioppo *et al*., 2015). However, the association between loneliness and positive emotions still remains largely unclear. Elucidating the relationships between loneliness and both positive and negative emotions is fundamental to building an affective framework of loneliness, which conceptualizes the development of loneliness based on both reward and punishment systems. This knowledge may then inform interventions targeted at reducing feelings of loneliness.

The association between high loneliness and negative emotions has been long established (Masi *et al*., 2011; Cacioppo *et al*., 2014). Previous research showed that negative and depressive emotions were longitudinally and positively related to high loneliness levels (Cacioppo *et al*., 2010). For example, according to a 5-year longitudinal study, loneliness prospectively predicted future onset of depressive symptoms (Cacioppo *et al*., 2010). Moreover, in a longitudinal study, it was discovered that among adults aged 50 years and older, elevated baseline loneliness scores were prospectively associated with greater severity of depressive symptoms at 12-year follow-up (Lee *et al*., 2021). Consistent with these findings, a negative emotion model of loneliness was previously proposed, which posited that individuals with high loneliness levels tend to exhibit automatic negative biases towards social interactions and relationships (Hawkley and Cacioppo, 2010). As a consequence, despite desiring social connections, these individuals choose to avoid and withdraw from social occasions, ultimately becoming socially isolated (Holt-Lunstad *et al*., 2015; Bzdok and Dunbar, 2020). This model is supported by existing evidence indicating high social anxiety levels among lonely individuals (Maes *et al*., 2019; Lieberz *et al*., 2022).

**Figure 1:**
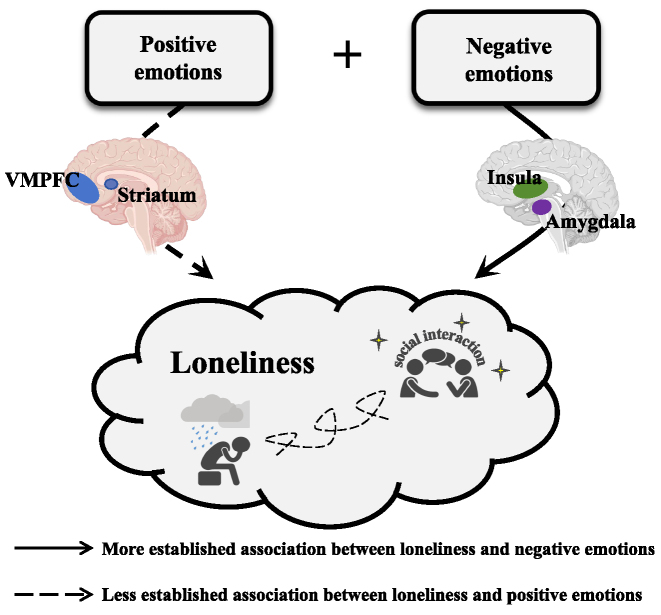
The bivalence emotion model of loneliness. The bivalence emotion model of loneliness suggests that changes in both positive and negative emotion systems can lead to loneliness. Furthermore, different brain regions are involved in processing various emotional aspects of loneliness. For instance, the striatum and VMPFC handle the positive emotions, while the insula and amygdala are responsible for processing the negative emotions. Existing evidence indicates a closer connection between loneliness and negative emotions, whereas the connection to positive emotions is less clear. This figure was created using materials obtained from BioRender.com, with full license granted.

On the other hand, the relationship between loneliness and positive emotions has received less attention. It is well-established that human decisions and behaviours are governed by both negative and positive emotion systems (Cardi *et al*., 2015). Thus, it is reasonable to presume that loneliness also has both positive and negative emotion components (Fig. 1). Previous work indicated that lonely individuals may exhibit blunted positive emotions, which is linked with a reduced capacity to perceive positive cues and enjoy positive experiences within social settings (Lieberz *et al*., 2021). According to this view, loneliness arises from a combined influence of reduced positive emotions and heightened negative emotions, particularly in social contexts (i.e. a dual emotion model). On the other hand, it could also be that lonely individuals show normal levels of positive emotional processing (Wong *et al*., 2019a), yet they withdraw from social interactions due to excessive negative emotions, as outlined before (i.e. a negative emotion model). To our knowledge, existing literature has not provided a direct answer to these alternative frameworks.

While previous evidence on the neuroimaging mechanisms of loneliness is limited (Wong *et al*., 2022), it is likely that loneliness is linked to altered functioning of brain circuitries involved in emotion processing, emotion regulation, and social cognition functions. Based on extensive previous literature, the positive emotion system includes the ventromedial prefrontal cortex (VMPFC) and the striatum (Doré *et al*., 2017). The negative emotion system includes the amygdala and insula (Shiba *et al*., 2017). The emotion regulation system includes the lateral prefrontal cortex (LPFC) (Hooker *et al*., 2010; Townsend *et al*., 2013), the anterior cingulate cortex (ACC) (Ochsner *et al*., 2012), and the hippocampus (Doré *et al*., 2018; Barch *et al*., 2020), whereas the social cognition system includes the dorsomedial prefrontal cortex (DMPFC) (Eickhoff *et al*., 2016). Among these regions, the striatum and VMPFC are mainly responsible for positive emotional processing (Hiser and Koenigs 2018; Filimon *et al*., 2020), the amygdala and insula are mainly involved in negative emotional processing (Goldin *et al*., 2008; Shiba *et al*., 2017; Steward *et al*., 2022), the LPFC and hippocampus are involved in top-down emotion regulation, and the DMPFC is involved in social cognition (Townsend *et al*., 2013; Lieberman *et al*., 2019; Arbula *et al*., 2021). These systems have been explained by extensive literature that largely agrees on the core brain circuitries involved in emotion reactivity (positive and negative), emotion regulation, and social processing (e.g. Ochsner *et al*., 2012; Roy *et al*., 2012; Etkin *et al*., 2015; Whittaker *et al*., 2018; Ferrari *et al*., 2016; Martin *et al*., 2017). Nevertheless, it should be noted that any categorization of regions into functional systems is inevitably subject to contention, and these systems merely serve as a provisional guide for our review of the loneliness-related neuroimaging literature rather than implying definitive functions of any brain region. As of now, it is still unclear how past neuroimaging studies on loneliness can inform whether loneliness is associated with altered functions of both positive and negative emotion systems, or whether it is primarily related to negative emotional function changes.

Therefore, this systematic review synthesized previous behavioural and neuroimaging studies on the association between loneliness and both positive and negative emotions to clarify the difference between the 'dual emotion model' and the 'negative emotion model' frameworks of loneliness. In the present context, loneliness levels were measured by different versions of the UCLA loneliness scale (ULS). The original version of the ULS comprises 20 items with four-point Likert scales, and assesses the individual's perceived subjective feelings of social isolation (Russell, 1996). Subsequent research has validated short versions of the ULS such as ULS-8, ULS-6, and ULS-3 across populations and cultures (Hays and DiMatteo, 1987; Russel 1996; Hughes *et al*., 2004; Neto, 2014; Lin *et al*., 2022).

## Methods

We conducted this review following the Preferred Reporting Items for Systematic Reviews and Meta-Analyses (PRISMA) guidelines (Page *et al*., 2021). This review protocol was pre-registered at PROSPERO, International prospective register of systematic reviews (reference number CRD42023445805). The PRISMA checklist can be found in Supplementary Table S1.

### Search strategy and study selection

**Figure 2:**
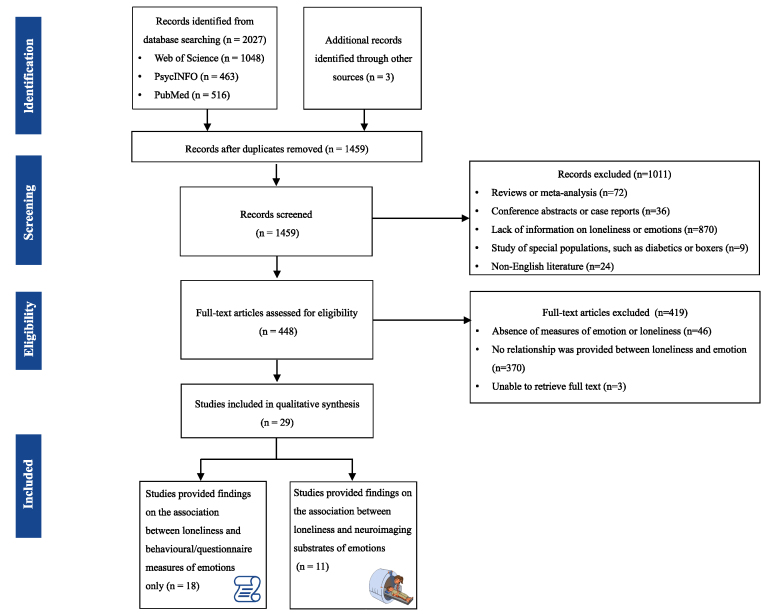
PRISMA flowchart.

We used the following key terms to search the MEDLINE/PubMed/PsycINFO online database to identify pertinent articles published up to 4 May 2023: (lonel*) AND ('positive emotion*' OR 'negative emotion*' OR 'positive affect*' OR 'negative affect*' OR reward* OR punishment* OR reinforcement*). Moreover, we conducted a comprehensive review of the reference lists of relevant articles to identify additional studies. The inclusion of identified studies was verified by two authors (R.S. and Q.L.). The entire literature selection process is depicted in Fig. 2. We only included studies that met the following criteria: (i) use of the ULS (ULS-20, ULS-3, ULS-6, ULS-8) to assess loneliness (Hays and DiMatteo, 1987; Russel 1996; Hughes *et al*., 2004; Neto, 2014), (ii) explicit measurement of positive and/or negative emotions using scales (e.g. the Positive and Negative Affect Schedule or PANAS, Watson *et al*., 1988; Beck's Depression Inventory or BDI, Beck *et al*., 1988) or task paradigms, (iii) publication in English, (iv) inclusion of at least 10 human participants (Lam *et al*., 2021), and (v) provision of quantitative results on the relationship between loneliness and emotions. Animal studies, literature reviews, and conference abstracts were excluded. We also excluded studies involving samples of special characteristics (e.g. military soldiers) or major physical illnesses (diabetes) (Supplementary Table S2).

The search resulted in a total of 2027 articles of interest (1048 articles from Web of Science, 463 articles from PsycINFO, and 516 from PubMed). After eliminating 568 duplicate articles, in the first stage of screening, the titles and abstracts of the remaining 1459 studies were evaluated by two authors (Q.L. and R.S.). Studies that met any of the exclusion criteria as outlined before were screened out. A total of 1011 studies were excluded during the first stage of screening. The full text of the remaining 448 studies was further assessed to resolve any remaining uncertainty. Disagreements during this phase were resolved through discussions between the two authors (Q.L. and R.S.). Finally, 29 studies published between 2010 and 2023 were chosen to be reviewed in this study (Fig. 2). Among these, 18 studies provided findings only on the association between loneliness and behavioural/questionnaire measures of emotions, 11 studies provided findings only on the association between loneliness and neuroimaging substrates of emotions only, and six studies provided findings on the association between loneliness and both behavioural/questionnaire and neuroimaging measures of emotions.

### Data extraction

Data extraction was independently performed by two authors (Q.L. and R.S.). For each eligible article, we extracted the following information: author(s), year of publication, participant characteristics (diagnosis, age, sex ratio, and sample size), mood measurement, loneliness measurement, and main findings (Tables 1 and 2).

**Table 1:** Summary of studies that tested the association of questionnaire measures of emotion and loneliness.

Study	Sample size	Sex ratio (F%)	Age (mean; SD/range)	Emotion	Emotion measure	Loneliness measure	Main findings	Quality assessment
**Negative emotion**								
Hoffman *et al*., 2021	120	40%	70.31 ± 5.93	Social anxiety (past week)	LSAS	20-item UCLA	Loneliness level was positively correlated with social anxiety in older adults.	7
Mikocka-Walus *et al*., 2021	2110	80.60%	38.25 ± 7.07	Depressive, anxiety and stress symptoms (past week)	DASS	6-item UCLA	Loneliness level was associated with stress, but not depressive or anxiety symptoms, during the COVID-19 pandemic.	8
Velotti *et al*., 2021	1323	77%	35.38 ± 14.08	Depression, anxiety, and stress symptoms (past week)	DASS-21	20-item UCLA	Loneliness level was positively correlated with depression, anxiety, and stress emotions.	8
Wegner & Liu, 2023	1354	75.50%	35.3 ± 12.9	Distress emotions due to COVID-19 (past 1–2 weeks)	CPDI	8-item UCLA	Loneliness level was positively associated with distress emotions in response to the COVID-19 pandemic.	13
Shi *et al*., 2023	4191	45.60%	19.14 ± 1.02	Depressive symptoms (past 2 weeks)	PHQ-9	8-item UCLA	Loneliness level was positively correlated with depressive symptoms.	13
Tan *et al*., 2020	824	72.30%	21.03 ± 5.59	Social anhedonia (long term)	RSAS	20-item UCLA	Loneliness level was positively correlated with social anhedonia level.	8
**Positive emotion**								
Davidson *et al*., 2022	428	48%	>60	Happiness level (past week)	CES-D	20-item UCLA	Loneliness level was negatively correlated with happiness level in older adults.	7
Donizzetti and Capone 2023	1301	56.10%	77.25 ± 5.46	Well-being (last month) General positive and negative emotions (past week)	MHC-SF, PANAS	20-item UCLA	Loneliness level was negatively correlated with positive emotions and with well-being.	11
Ben-Zur 2012	196	54%	45.94 (mean age)	Dispositional optimism	LoT, PANAS-N	20-item UCLA	Loneliness level was negatively correlated with optimism and positively correlated with negative emotions.	7
**Both positive and negative emotions (general)**								
Ditcheva *et al*., 2018	118	38.10%	18.7 ± 0.89	General positive and negative emotions (at baseline and after acute stress induction)	PANAS	20-item UCLA	Loneliness was negatively correlated with positive emotions at baseline. After acute stress induction using a modified Trier Social Stress Test, loneliness was associated with greater increase of positive emotions under the stress condition, but smaller increase of positive emotions under the no-stress control condition.	14
Satici 2019	280	57.50%	21.04 ± 1.43	General positive and negative emotions (past week)	PANAS	8-item UCLA	Loneliness level was negatively correlated with positive emotions and positively correlated with negative emotions in adults.	7
Neto 2014	1154	60.50%	71.26 ± 6.66	General positive and negative emotions (past week)	PANAS	6-item UCLA	Loneliness level was negatively correlated with positive emotions and positively correlated with negative emotions.	7
Baytemir & Yildiz, 2017	263	59%	15.05 ± 0.90	General positive and negative emotions (past week)	PANAS, SASA, CDI	8-item UCLA	Loneliness level was negatively correlated with positive emotions and positively correlated with anxiety and depressive symptoms.	9
Durak & Senol-Durak, 2010	481	60.90%	21.57 ± 1.92	General positive and negative emotions (past week)	BDI、PANAS	20-item UCLA	Loneliness level was negatively correlated with positive emotions and positively correlated with negative emotions.	10
Serra *et al*., 2021	61	62.30%	82.77 ± 7.779	General positive and negative emotions (past week)	PANAS	20-item UCLA	Loneliness level was negatively correlated with positive emotions, and positively correlated with negative emotions	8
**Both positive and negative emotions (momentary)**								
Tang *et al*., 2022	177	61%	19.90 ± 2.76	Momentary positive and negative emotions	ESM	20-item UCLA	Loneliness level was negatively correlated with positive emotions in adolescents.	7
Badal *et al*., 2022	22	86.40%	80.24 ± 7.13	Momentary positive and negative emotions	EMA	20-item UCLA	Loneliness level was positively correlated with momentary negative emotions.	10
Steptoe *et al*., 2011	4258	55.10%	64.3 (mean age)	Momentary positive emotions, depressive symptom (past week)	EMA, CES-D	3-item UCLA	Loneliness level was negatively correlated with momentary positive emotions, and positively correlated with depressive symptoms in older adults.	10

Abbreviation: BDI: Beck Depression Inventory; CDI: Children's Depression Inventory; CES-D: Center for Epidemiologic Studies Depression Scale; COVID-19: Coronavirus Disease 2019; CPDI: COVID-Peritraumatic Distress Index; DASS: Depression Anxiety and Stress Scale; DASS-21: Depression Anxiety and Stress Scale-21 items; ESM: Experience Sampling Method; EMA: Ecological momentary assessment; LSAS: Leibowitz Social Anxiety Scale; LoT: Life Orientation Test; MHC-SF: Mental Health Continuum-Short Form questionnaire; PANAS-N: PANAS—Expanded Form; PHQ-9: Patient Health Questionnaire-9; RSAS: Revised Social Anhedonia Scale; SASA: Revised Social Anhedonia Scale.

**Table 2:** Summary of neuroimaging studies which tested the association of emotion and loneliness.

Study	Sample size	Sex ratio	Age (mean; SD/range)	Emotion measured	Tool of measurement	Loneliness measure	Statistical threshold	Main finding	Quality assessment
**Loneliness and the positive emotion system**									
Sin *et al*., 2018	52	63%	67.92 ± 5.0	Depressive patients vs. healthy controls	Clinical diagnosis	20‐item UCLA	*P*_corr_ < 0.01	Among older adults, loneliness level was higher in depressive patients, and showed a positive correlation with the number of episodes. Loneliness level was positively associated with left striatal grey matter volume among single-episode depressive patients, while the relationship was reversed for multiple-episode depressive patients.	7
Inagaki *et al*., 2016	31	48.30%	24.26 ± 7.57	PA (current state)	Imaging task	20‐item UCLA	*P* < 0.05, two-tailed	Loneliness level was negatively correlated with feelings of connection with close others in healthy young adults, and loneliness was negatively correlated with higher ventral striatal activity when viewing images of close people versus strangers.	9
***Loneliness and the negative emotion system***									
Wong *et al*., 2016	31	54.80%	67.45 ± 5.42	Brain region of negative emotion system (amygdala)	N/A	20‐item UCLA	*P*_FDR_ < 0.05	Loneliness was negatively associated with the edges between the amygdala and superior frontal gyrus in white-matter structural network.	10
Wong *et al*., 2019b	40	55%	17.8 ± 1.21	Positive and negative emotions (in general)	20-item Chinese Affect Scale	20‐item UCLA	*P*_Bonferroni_ < 0.05	Loneliness level was negatively associated with sibling-average trait positive affect, and positively associated with sibling-average trait negative affect. Sibling-average trait negative affect was positively associated with local efficiency of structural network in the insula and the LPFC, which contributed to mediating the positive relationship between loneliness and trait negative affect.	10
**Loneliness and the emotional regulation system**									
Tian *et al*., 2017	30	0%	21.3 ± 2.4	Brain region of emotion regulation system (inferior frontal gyrus, superior frontal gyrus)	N/A	20‐item UCLA	*P*_FDR_ < 0.05	Loneliness was associated with weaker top-down control flow from the dorsal attentional network (including parts of the inferior frontal gyrus, the superior frontal gyrus) to the ventral attentional network (including parts of the inferior frontal gyrus).	10
Ohtsubo *et al*., 2020	26	88.50%	20.35 ± 1.44	General positive and negative emotions (current state)	Task-based MRI	20‐item UCLA	*P*_FWE_ < 0.05	High loneliness level was associated with lower VMPFC activity in response to low-cost commitment signals on a social exchange evaluation task.	10
Shao *et al*., 2020	27	77%	51.85 (mean age)	General positive and negative emotions (current state)	Resting-state MRI	20‐item UCLA	*P*_FDR_ < 0.05	Loneliness level was associated with lower resting-state functional connectivity between parietal and anterior cingulate in MDD patients.	14
Dong *et al*., 2021	57	49.10%	20.88 ± 2.093	Brain region of emotion regulation system (left DLPFC)	N/A	20‐item UCLA	voxel: *P*_GRF_ < 0.001 cluster: *P*_GRF_ < 0.05	Among internet gaming addicts, loneliness was associated with reduced resting-state functional connectivity between the left DLPFC and a sensorimotor network, among a group of internet gaming addicts.	13
Liu *et al*., 2016	405	54%	19.94 ± 1.19	Brain region of emotion regulation system (left DLPFC)	N/A	20‐item UCLA	*P*_AlphaSim_ < 0.05	Loneliness level was associated with increased gray matter volume in the left DLPFC.	12
**Loneliness and the positive emotion system and the emotional regulation system**									
Lieberz *et al*., 2022	82	50%	26.83 ± 7.47	Social anxiety (past week)	LSAS	20‐item UCLA	*P*_FWE_ < 0.05	In healthy young adults, loneliness was positively associated with social anxiety level. In a social gambling paradigm, high-loneliness participants showed more pleasant ratings to negative human feedback, and reduced striatal activity but enhanced striatum-hippocampus connectivity to negative human feedback relative to computer feedback, compared to low-loneliness participants.	11
**Loneliness and the positive emotion system, the emotional regulation system amd the social cognition system**									
Cacioppo *et al*., 2009	23	100%	young adults	Neural processing of positive and negative emotional stimuli	Emotion processing Task	20‐item UCLA	voxelwise threshold of *P* < 0.025	Loneliness level was negatively associated with ventral striatal and DMPFC activities to pleasant social vs. pleasant non-social stimuli, and negatively associated with right ventrolateral prefrontal cortex activities to unpleasant social vs. unpleasant non-social stimuli.	9

Abbreviation: Corr: correction; FDR: false discovery rate; FWE: family-wise error; GRF: Gaussian random field; LSAS: Leibowitz social anxiety scale; MDD: major depressive disorder; MRI: magnetic resonance imaging; PA: positive affect.

### Quality assessment

The articles that met the eligibility criteria were assessed on quality using the BIOCROSS evaluation tool (Wirsching *et al*., 2018), specifically designed to evaluate the quality of biomarker-based cross-sectional studies. The BIOCROSS tool comprises ten items grouped into five domains: 'Study rationale', 'Design/Methods', 'Data analysis', 'Data interpretation', and 'Biomarker measurement'. Each item has three 'issues to consider' that evaluate the quality of the study, and scores of 0, 1, and 2 are assigned depending on the number of issues reported. On the basis of the obtained score, the quality of the studies was classified as low, moderate, or high, with scores of ≤6 indicating low quality, scores of 7–12 indicating moderate quality, and scores of ≥13 indicating high quality. The results of the quality assessment are presented in Tables 1 and 2 and Supplementary Table S3.

## Results

### Characteristics of included studies

Out of 2027 records in total, 29 papers were included in this systematic review. Eighteen studies provided only behavioural results (four tested the relationship between loneliness and positive emotion, seven tested the relationship between loneliness and negative emotion, seven tested the relationship between loneliness and both positive and negative emotions). Five studies provided only neuroimaging results (one tested the relationship between loneliness and positive emotions, one tested the relationship between loneliness and negative emotions, three tested the relationship between loneliness and both positive and negative emotions). Six studies provided both behavioural and neuroimaging results (one tested the relationship between loneliness and positive emotions, two tested the relationship between loneliness and negative emotions, three tested the relationship between loneliness and both positive and negative emotions). Among the 11 studies that provided neuroimaging results, two studies reported only the participants' gray matter measurements, one study reported only the participants' white matter connectivity, four studies reported participants' task-based activity and connectivity, three studies reported participants' resting-state functional connectivity, and one study reported both white matter and task-based connectivity.

The studies' sample sizes ranged from 22 to 4258 participants. The total sample size was 19 465 (female% = 59.8%). Among the 29 studies, 12 (41.3%) involved <100 participants, 10 (34.4%) included 100–1000 participants, and seven (24.1%) included >1000 participants. The age range was 14–91 years (mean = 38.42 years, SD = 23.59 years). Of the 29 included studies, two studied adolescents (<18 years), 11 studied young adults (18–24 years), seven studied adults (25–60 years), and nine studied the older population (>60 years). Twenty-six studies examined psychologically healthy individuals and three studies examined depressed patients.

### Study quality assessment

The BIOCROSS tool was used to assess the quality of the 29 studies. Among these, five studies were rated as high quality (≥13 score), while 24 studies were rated as having moderate quality (7–12 score). No study was rated as low quality (<7 score).

### The behavioural association between loneliness, and positive and negative emotions

#### Positive emotions

A total of 10 studies investigated the association between loneliness and positive emotions. Among these, one study was conducted in adolescents (Baytemir and Yildiz, 2017), four studies were conducted in young adults (Durak and Senol-Durak, 2010; Ditcheva *et al*., 2018; Satici 2019; Tang *et al*., 2022), one was conducted in middle-aged adults (Ben-Zur 2012), and four were conducted in older adults (Steptoe *et al*., 2011; Neto 2014; Davidson *et al*., 2022; Donizzetti and Capone 2023). All 10 studies reported a negative relationship between loneliness and positive emotion among healthy individuals, although one study additionally found that this relationship may be reversed following social stress (as detailed next).

Among adolescents, Baytemir and Yildiz (2017) (*n* = 263, mixed-sex) showed that loneliness as measured using the ULS-8 was negatively associated with general positive emotions as measured using the Positive and Negative Affect Scale (PANAS). This was the only study in adolescents that tested and demonstrated that loneliness was associated with reduced general positive emotions.

Among young adults, two studies also reported a negative relationship between loneliness as measured using the ULS-8 (Satici 2019, *n* = 280, mixed-sex) or the ULS-20 (Durak and Senol-Durak, 2010, *n* = 481, mostly females), and general positive emotion as measured using the PANAS. A third study (Tang *et al*., 2022, *n* = 177, mostly females) measured participants’ momentary positive emotions, and also found that loneliness (measured using ULS-20) was negatively associated with positive emotions such as excitedness and cheerfulness. The fourth study measured participants’ momentary positive emotions both at baseline and after acute stress induction using the Trier Social Stress Test (Ditcheva *et al*., 2018, *n* = 196, mixed-sex). This study again found that loneliness (measured using ULS-20) was negatively associated with momentary general positive emotion as measured using the PANAS. However, it was further found that following acute stress, lonelier individuals exhibited a greater increase of positive emotions compared to less lonely individuals, whereas loneliness was associated with less increase of positive emotions following the no-stress control condition. This finding highlights an intricate interplay between trait loneliness and situational social appraisal on position emotional states.

Among middle-aged adults, one study (Ben-Zur 2012, *n* = 196, mixed-sex) used a sample of married, divorced, and widowed individuals, and showed that loneliness as measured using the ULS-20 was negatively associated with dispositional optimism.

Among older adults, similar to the previous results in younger samples, two studies both showed that loneliness as measured using the ULS-6 (Neto 2014, *n* = 1154, mostly females) or the ULS-20 (Donizzetti and Capone 2023, *n* = 1301, mixed-sex) was negatively associated with general positive emotions as measured using the PANAS. The latter study additionally revealed a negative relationship between participants’ loneliness level and past-month wellbeing. Moreover, a large-sample study (Steptoe *et al*., 2011, *n* = 4258, mixed sex) reported a negative correlation between loneliness as measured using the ULS-3, and momentary positive emotions such as happiness and excitedness. Last, one recent study (Davidson *et al*., 2022, *n* = 428, mostly males) showed that loneliness measured using the ULS-20 was negatively associated with past-week happiness level.

### Summary

Overall, past studies generated consistent results that loneliness was associated with reduced positive emotions in both sexes. The sample sizes of the studies were mostly large. Most studies examined general positive emotions using the PANAS scale, while a small number of studies examined specific positive emotions such as happiness, excitedness, and life satisfaction. Most studies examined positive emotions within the past week, while the remaining studies looked at momentary or dispositional positive emotions. However, all the existing studies were cross-sectional, thus the direction of the loneliness–positive emotion relationship remains unclear. Also, there was a paucity of studies that mined specific positive emotions, particularly positive social emotions such as a feeling of unity and connectedness. Furthermore, it remains to be tested how the relationship between loneliness and positive emotions may depend on situational factors such as the social environment and stress level.

#### Negative emotions

A total of 14 studies investigated the association between loneliness and negative emotions. Among these, one study was conducted in adolescents (Baytemir and Yildiz, 2017), four studies were conducted in young adults (Durak and Senol-Durak, 2010; Satici 2019; Tan *et al*., 2020; Shi *et al*., 2023), four were conducted in middle-aged adults (Ben-Zur 2012; Velotti *et al*., 2021; Mikocka-Walus *et al*., 2021; Wegner and Liu, 2023), and five were conducted in older adults (Steptoe *et al*., 2011; Neto 2014; Serra *et al*., 2021; Hoffman *et al*., 2021; Badal *et al*., 2022). All 14 studies reported a positive relationship between loneliness and negative emotions among healthy individuals.

Among adolescents, Baytemir and Yildiz (2017) (*n* = 263, mixed-sex) showed that loneliness as measured using the ULS-8 was positively associated with anxiety and depressive symptoms as measured using the Social Anxiety Scale for Adolescents and Children's Depression Inventory, respectively. This was the only study in adolescents that tested and demonstrated that loneliness was associated with increased anxiety and depression.

Similarly, in young adults, two studies found that loneliness as measured using the ULS-20 or ULS-8 was positively correlated with depressive symptoms in the past 2 weeks (Durak and Senol-Durak, 2010; *n* = 481, mostly females; Shi *et al*., 2023, *n* = 4191, mixed-sex). Moreover, one study reported a positive correlation between loneliness as measured using ULS-20, and social anhedonia level (Tan *et al*., 2020, *n* = 824, mostly females). Last, one study showed a positive correlation between loneliness as measured using ULS-8, and general negative emotions in the past week (Satici 2019, *n* = 280, mixed-sex).

In middle-aged adults, it was again showed that loneliness as measured using the ULS-20 was positively correlated with depression, anxiety, and stress symptoms (Velotti *et al*., 2021; *n* = 1323, mostly females). However, another study only reported a significant positive correlation between loneliness as measured using ULS-6 and stress symptoms, but not with depression or anxiety symptoms (Mikocka-Walus *et al*., 2021, *n* = 2110, mostly females). The difference in findings might be caused by the latter study specifically testing the relationship between loneliness and negative symptoms under the COVID-19 pandemic. Furthermore, one study showed that loneliness as measured using ULS-20 was positively correlated with general negative emotions (Ben-Zur 2012; *n* = 196, mixed-sex), and another study showed that loneliness as measured using ULS-8 was positively associated with distress emotions to the COVID-19 pandemic (Wegner and Liu, 2023, *n* = 1354, mostly females).

Among older adults, similar to the findings in younger samples, it was shown that loneliness as measured using ULS-3 was positively correlated with depressive symptoms in the past week (Steptoe *et al*., 2011; *n* = 4258, mixed-sex), while loneliness as measured using UCLA-20 was positively correlated with social anxiety level in the past week (Hoffman *et al*., 2021, *n* = 120, mixed-sex). Two other studies both found that loneliness as measured using ULS-6 or ULS-20 was positively associated with general negative emotions in the past week (Neto 2014, *n* = 1154, mixed-sex; Serra *et al*., 2021, *n* = 61, mixed-sex). Last, one study reported that loneliness as measured using ULS-20 was positively associated with momentary negative emotions (Badal *et al*., 2022, *n* = 22, mostly females).

### Summary

A considerable body of evidence supports a positive relationship between loneliness and negative emotions, particularly in adults and older individuals. Most of the studies have included relatively large sample sizes (*n* > 200). Whereas many studies included samples more representative of females, several studies used mixed-size samples and obtained similar findings. The association between loneliness and (dis)stress emotions was consistently reported, while the association between loneliness, depression, and anxiety symptoms was mostly found. Notably, while almost all studies assessed relatively short-term negative moods or symptoms, one study (Tan *et al*., 2020) reported a significant positive correlation between loneliness and long-term social anhedonia, which indicates that loneliness may be linked with a dispositional depressive trait. It is also worth noting that there was no study that measured negative emotions using behavioural paradigms, which could be a direction for future studies. Moreover, the lack of longitudinal or intervention studies precluded any inferences on the direction of causality.

### The association of loneliness and emotion-related brain circuitries

A total of 11 studies examined the association between loneliness and brain circuitries associated with emotional functions. All these studies measured loneliness using ULS-20. The positive emotion system includes the VMPFC and the striatum (Doré *et al*., 2017). The negative emotion system includes the amygdala and insula (Shiba *et al*., 2017). The emotion regulation system includes the LPFC and the hippocampus (Hooker *et al*., 2010; Townsend *et al*., 2013), while the social cognition system includes the DMPFC (Townsend *et al*., 2013; Lieberman *et al*., 2019; Arbula *et al*., 2021; Li *et al*., 2022). Within the 11 studies, four studies examined the correlates of loneliness and the positive emotion system (Cacioppo *et al*., 2009; Sin *et al*., 2018; Inagaki *et al*., 2016; Lieberz *et al*., 2022), whereas two articles investigated the correlates between loneliness and the negative emotion system (Wong *et al*., 2016; Wong *et al*., 2019b). Furthermore, seven studies tested the correlates of loneliness and the emotional regulation system (Cacioppo *et al*., 2009; Tian *et al*., 2017; Ohtsubo *et al*., 2020; Shao *et al*., 2020; Dong *et al*., 2021; Lieberz *et al*., 2022; Liu *et al*., 2016), whereas one study tested the correlates of loneliness and the social cognition system (Cacioppo *et al*., 2009). It should be noted that we were unable to review the relationship between loneliness and certain key regions involved in emotion regulation functions, such as the parietal cortex, because existing studies did not report findings on those regions. It is also very important to note that while we based the review of the neuroimaging findings on the proposed emotion systems, one should always be cautious when making inverse inferences about psychological processes from neuroimaging findings.

#### Positive emotion system

Five studies examined the relationship between loneliness and the positive emotion system. Four of these studies employed the task-based fMRI design (Cacioppo *et al*., 2009; Inagaki *et al*., 2016; Ohtsubo *et al*., 2020; Lieberz *et al*., 2022), while the fifth study measured participants’ grey matter volume (Sin *et al*., 2018).

Four studies were conducted in healthy young adults. One recent study (Lieberz *et al*., 2022*, n* = 82, mixed-sex) discovered that higher loneliness level was correlated with greater social anxiety as assessed using the Liebowitz Social Anxiety Scale. In this study, participants who were classified as either high- or low-loneliness performed a social gambling paradigm in which their gambling choices were followed by either human or computer feedback. The findings showed that high-loneliness participants gave more pleasant ratings to negative human feedbacks, and showed reduced striatal activity but enhanced striatum-hippocampus functional connectivity when receiving negative human versus computer feedbacks, compared to low-loneliness participants. Another study (Cacioppo *et al*., 2009, *n* = 23, all females) showed that loneliness level was negatively related to ventral striatal activities when viewing pleasant social versus pleasant non-social pictorial stimuli. A third study (Inagaki *et al*., 2016, *n* = 31, mixed-sex) showed that lonelier individuals reported reduced feelings of connection with close others, and higher ventral striatal activity when viewing images of close others versus strangers. Last, Ohtsubo *et al*. (2020) (*n* = 22, mostly females) examined the role of the VMPFC function in loneliness. This study administered a 'commitment signaling task', which measured participants’ evaluation of social exchange signals. This study found that high loneliness was associated with lower activity in the VMPFC when evaluating low-cost commitment signals. This result indicates that lonely individuals may derive less pleasure from relatively ambiguous social exchange signals.

The fifth study tested the relationship between loneliness level and grey matter volume in older depressed patients (Sin *et al*., 2018, *n* = 52, mixed-sex). They found that loneliness level was positively associated with left striatal grey matter volume in single-episode patients, but the relationship was reversed among multiple-episode patients (i.e. negative relationship). In this study, patients with multiple episodes on average had experienced 2.7 depression episodes, while single-episode patients had experienced only one depression episode. The moderation of loneliness-striatal volume relationship by depression episode number may reveal progressive depression-related brain pathology that affects the coping mechanism of the positive emotion system to loneliness traits. It was previously proposed that the association between loneliness and brain patterns may show a nonlinear trajectory as lonely individuals progressively become increasingly depressed, such that upregulation of neural structure or function may manifest during earlier stage of depression that represent compensatory processes to the negatively biased emotions (Shao *et al*., 2020). However, such upregulation may reverse direction as the compensatory process becomes exhausted in the long term among patients with multiple depression episodes that are known to cause neurotoxicity and cellular deterioration (Belleau *et al*., 2019).

Collectively, the existing evidence generally indicates a negative relationship between loneliness and the functional activity of the brain positive emotion system including the striatum and the VMPFC. However, the findings were not entirely consistent regarding the sign of the relationship (Inagaki *et al*., 2016). Also, findings on the relationship between loneliness and structural volume of the positive emotion system were still lacking and inconclusive. In general, evidence is lacking for healthy middle-aged and older adults. Moreover, the sample sizes of existing studies were mostly modest, and only cross-sectional observational data were available.

#### Negative emotion system

Two studies examined the relationship between loneliness and the negative emotion system. One of these studies employed the task-based fMRI design (Wong *et al*., 2016), whereas the other study (Wong *et al*., 2019b) constructed structural brain networks for graph theory analysis. Among these studies, one (Wong *et al*., 2019b) looked at the role of insula in the negative emotion system and the other one (Wong *et al*., 2016) studied the involvement of the amygdala.

##### Insula

Among adolescents, Wong *et al*., (2019b) (*n* = 40, mixed-sex) measured the positive and negative trait affect of siblings via the 20-item Chinese Affect Scale, and found that loneliness level was negatively related to sibling-average positive affect, and positively related to sibling-average negative affect. In addition, the siblings’ mean negative affect levels were positively correlated with local efficiencies of white-matter networks in the insula. Furthermore, the local structural network efficiency of the insula contributed to mediating the relationship between loneliness and trait negative affect. These findings supported the relationships of loneliness and both positive and negative trait affect, and implicated the insula as a core region of the negative emotion system where structural network changes may underpin the association between loneliness and increased negative emotion levels. However, given this was a cross-sectional study, the causal relationship could not be established.

##### Amygdala

Among depressed and healthy older adults, Wong *et al*. (2016) (*n* = 31, mixed-sex) looked at the role of the amygdala in the negative emotional systems associated with loneliness. This study examined the relationship between loneliness and white-matter structural network connectivity strengths, and found that loneliness was negatively associated with the connectivity between the amygdala and the superior frontal gyrus. This finding indicated that loneliness could be linked with reduced regulation of the amygdala by dorsal prefrontal regions, which may result in amygdala hyperactivity and increased negative emotion bias.

In summary, limited existing evidence supports a link between loneliness and the white-matter structural networks of the negative emotion system, particularly regarding the insula and amygdala. It is possible that higher local efficiency but reduced regulatory connectivity from the dorsal prefrontal cortex collectively result in enhanced activity of the negative emotion system in lonelier individuals. However, the evidence is too limited to draw a conclusion, and no causal relationship could be established.

#### Emotion regulation system

A total of seven studies explored the relationship between loneliness and emotion regulation systems. Of these studies, one was conducted in adolescents (Wong *et al*., 2019b), four in young adults (Cacioppo *et al*., 2009; Tian *et al*., 2017; Dong *et al*., 2021; Liu *et al*., 2016), one in healthy adults (Lieberz *et al*., 2022), and one in MDD patients (Shao *et al*., 2020). Among these studies, five studies (Cacioppo *et al*., 2009; Tian *et al*., 2017; Wong *et al*., 2019b; Dong *et al*., 2021; Liu *et al*., 2016) examined the role of the LPFC in emotion regulation. The sixth study (Lieberz *et al*., 2022) examined the contribution of the hippocampus in emotion regulation. The last study (Shao *et al*., 2020) looked at the involvement of the anterior cingulate cortex.

##### Lateral prefrontal cortex

Among the five studies that examined the association between loneliness and the LPFC, one examined task-based brain activities (Cacioppo *et al*., 2009), two examined resting-state functional connectivity (Tian *et al*., 2017; Dong *et al*., 2021), one examined brain grey matter volume (Liu *et al*., 2016), and one looked at participants’ white matter tracts (Wong *et al*., 2019b).

Cacioppo *et al*. (2009) (*n* = 23, all females) showed that loneliness level was negatively related with right ventrolateral prefrontal cortex activities during viewing unpleasant social versus unpleasant non-social pictorial stimuli. In the same vein, another study (Dong *et al*., 2021, *n* = 57, mixed-sex) found that among a sample of internet gaming addicts, higher loneliness was associated with reduced resting-state functional connectivity between the left dorsolateral prefrontal cortex (DLPFC) and cortical sensorimotor areas.

Moreover, Tian *et al*., (2017) (*n* = 30, all males) found that loneliness was associated with weaker resting-state top-down functional projection from the dorsal attentional network including the superior frontal gyrus and parts of the inferior frontal gyrus, to the ventral attentional network including parts of the inferior frontal gyrus (among others). Another study (Liu *et al*., 2016, *n* = 405, mixed-sex) investigated participants’ brain grey matter volume, and showed that high loneliness was associated with increased gray matter volume in the left DLPFC. The fifth study (Wong *et al*., 2019b, *n* = 40, mixed-sex) measured the trait affect of siblings via the 20-item Chinese Affect Scale, and found that the siblings’ mean negative affect levels were positively correlated with local efficiencies of white-matter networks in the LPFC, which contributed to mediating the relationship between loneliness and trait negative affect.

In summary, multimodal neural evidence consistently showed an association between loneliness level and LPFC structural and functional changes. Notwithstanding, the affective implication of these changes is unclear, with the only task-based imaging study indicating a relevance to negative social emotion regulation. Also, while functional imaging studies showed negative correlations between loneliness and LPFC activities and connectivity strengths, structural imaging studies showed positive relationships between loneliness and LPFC grey matter volume and white matter connectivity efficiency. The discrepancy in these findings could be due to structural compensatory processes in the emotion regulation network due to increased demand of regulating negative emotions among lonely individuals.

##### Hippocampus

One study described previously (Lieberz *et al*., 2022, *n* = 82, mixed-sex) discovered that high-loneliness participants showed enhanced striatum-hippocampus functional connectivity when receiving negative human versus computer feedback when performing a gambling task, compared to low-loneliness participants. The increase in functional connectivity between the striatum and the hippocampus may indicate potentiated negative emotion regulation using positive prospects among lonely individuals.

##### Anterior cingulate cortex

One study (Shao *et al*., 2020, *n* = 27, mostly females) showed that higher loneliness level was associated with increased resting-state functional connectivity between the anterior cingulate cortex and the inferior parietal cortex in depressed patients. This result again indicates potentiated regulation of the emotion control network (i.e. anterior cingulate cortex) on the default mode network (DMN) region (i.e. inferior parietal cortex) during spontaneous self-referential affective processing.

### Summary

Preliminary evidence from single studies indicates that in both healthy and depressed individuals, loneliness was positively associated with functional connectivity of the hippocampus and the anterior cingulate cortex with other brain regions involved in emotion and self-referential processing. This connectivity increase may represent potentiated efforts of lonely individuals in regulating negative emotions. These results are opposite to the decreased LPFC connectivity found in lonely participants as described earlier, which may depend on the precise brain circuitries, participant characteristics, and whether the connectivity was measured during task performance or resting state.

#### Social cognition system

Only one study (Cacioppo *et al*., 2009) explored the role of the DMPFC in the social cognition system related to loneliness. This study (*n* = 23, all females) showed that loneliness level was negatively related to DMPFC activities when viewing pleasant social versus pleasant non-social pictorial stimuli. This could indicate reduced social cognition and perspective taking in lonely individuals, particularly to positive social signals.

## Discussion

To the best of our knowledge, this was the first systematic review exploring a bidirectional emotional processing model of loneliness. A total of 29 studies meeting our research criteria indicated significant relationships between loneliness and the processing of positive and negative emotions. Behaviourally, a total of 10 studies consistently showed negative relationships between loneliness levels and positive emotions in general, but evidence was lacking for whether loneliness is associated with decreases in positive social emotions specifically. Position social emotions such as love, empathy, and social connectedness form the motivational bases for interpersonal relationships (Zaki, 2020), deficits in which are commonly observed in psychopathology (Blair, 2018). A total of 14 studies showed positive relationship between loneliness and negative emotions, which were exclusively obtained using questionnaire measures of negative emotions. Thus, it remained unclear whether loneliness is associated with negative emotions measured using behavioural paradigms. Furthermore, five studies that investigated the relationship between loneliness and the brain positive emotion system mostly reported a negative relationship, whereas limited evidence from only two studies reported a possibly positive relationship between loneliness and the brain negative emotion system such as the insula and the amygdala. However, the sign of the relationship (positive or negative) was not entirely consistent, and the sample sizes of those studies were relatively modest. Moreover, seven studies in total showed a consistent relationship between loneliness and the brain emotion regulation system, particularly in the prefrontal cortex, but the directional implication of the findings was unclear. Last, only one study reported a negative relationship between loneliness and functional activities of the brain social cognition system. One common shortcoming of the existing studies is the lack of longitudinal design, which precluded making causal inferences.

### The relationship between positive emotions and loneliness

Studies that examined the association between loneliness and questionnaire measures of positive emotions yielded consistent results with studies that examined the association between loneliness and brain circuitries implicated in positive emotion processing. Specifically, all the behavioural studies reported a significant negative relationship between self-reported loneliness level on one hand, and general or specific positive emotion on the other hand. In terms of the neuroimaging findings, most existing studies showed reduced functional activity in the positive emotion system (striatum and VMPFC) among lonely individuals, although there were also exceptions indicating a reverse (i.e. positive) relationship (Inagaki *et al*., 2016). Viewed together, it appeared that lonely individual's reduced positive emotion function might be due to their blunted functional level of the brain positive emotion system. Such a causal relationship needs to be directly tested by neuromodulation and longitudinal studies. Relevant to this, past research indicates that functional activity of the striatum and the VMPFC during a stressful experience predicted subsequent level of positive emotion state (Yang *et al*., 2018), and dampened the relationship between high life stress and low positive emotion state (Nikolova *et al*., 2012). Given loneliness is intimately related to high stress levels, it might be that reduced striatum and VMPFC activity renders lonely individuals more vulnerable to low positive emotion.

As noted before, one study reported a positive relationship between loneliness level and ventral striatal activity when viewing images of close others versus strangers (Inagaki *et al*., 2016). The reason for this result was unclear. The authors interpreted this finding as reflecting an increase in desire for social connectedness due to unmet social needs. It is possible that the sign of relationship (i.e. positive) reported in this study differed from that reported in the other studies (i.e. negative, Cacioppo *et al*., 2009; Ohtsubo *et al*., 2020; Lieberz *et al*., 2022) due to the nature of the task paradigm used. Inagaki *et al*. used a task involving viewing photographs of the participant's friend, but the task otherwise did not incorporate an explicit social interaction element. By contrast, the other studies all employed tasks involving non-personal stimuli with explicit social interaction processes. It could be that viewing photographs belonging to the participant's own friends elicited a greater desire for social interaction in lonely individuals compared to encountering social stimuli of strangers, which was reflected as increased ventral striatal activity (Inagaki *et al*., 2015).

It is also worth noting that in the study by Lieberz *et al*. (2022), lonely participants gave more pleasant ratings but showed lower striatal activity to negative human feedback when performing a gambling task. Given the task paradigm involved a risky decision-making process, it could be that the lower striatal activity represented reduced learning about negative social outcomes, given the important role of the striatum in reinforcement learning (e.g. Atallah *et al*., 2014). In this regard, it could be that lonely individuals showed a reduced capacity to utilize social information effectively to guide decision-making, which may arise from their difficulties in perceiving social cues (Kanai *et al*., 2012) or general perceptual decision-making (Mąka *et al*., 2023).

These studies had several limitations. First, the studies mostly included young and older adults, while few studies included adolescents and middle-aged adults. Emotion issues related to loneliness during adolescence and middle adulthood deserve special attention as loneliness is a prevalent experience during these life stages (Lee *et al*., 2019; Surkalim *et al*. 2022; Pengpid and Peltzer, 2021). Adolescence is a critical stage for establishing social and emotional styles that can influence mental well-being (Blakemore, 2019). During adolescence, the brain is highly plastic and undergoes rapid development, making it vulnerable to the influence of negative social environments (Lamblin *et al*., 2017; Vijayakumar *et al*., 2018). Similarly, emotions related to social relationships play a critical role in shaping the mental health of middle-aged adults (Oshio, 2014). Furthermore, brain network functions have been found to show considerable differences between adolescence and adulthood (Tian *et al*., 2016). For example, one study revealed significant changes in functional connectivity pattern of cortical networks such as the DMN from adolescence to middle adulthood (Tian *et al*., 2016). Therefore, it is important to study the relationship between loneliness and positive emotions, and the associated neural patterns, at each distinct developmental stage, particularly during adolescence and middle adulthood.

Another issue was that most existing research focused on examining the relationship between loneliness and positive emotions in general, while evidence was lacking on whether loneliness is associated with changes in positive social emotions specifically. Positive social emotions were defined as positive feelings related to social interactions and interpersonal relationships, typically involving experiences of connection, cooperation, friendship, love, and social support with others (Jankowski and Takahashi, 2014; Zaki, 2020; van Tilburg, 2023). While positive emotions in general and positive social emotions show certain correlations with each other, they have been found to dissociate from each other (Koush *et al*., 2019; Scharnowski *et al*., 2020; Lam *et al*., 2021; Atzil *et al*., 2023), and implicate only partially overlapping neural circuitries (Koush *et al*., 2019; Scharnowski *et al*., 2020). For example, while both positive emotions in general and positive social emotions implicate brain regions such as the striatum and DLPFC, the latter may additionally implicate the DMPFC and the subgenual anterior cingulate cortex (Koush *et al*., 2019; Scharnowski *et al*., 2020). Therefore, future studies should measure positive social emotions alongside positive emotions in general, using both questionnaires and task paradigms.

### The relationship between negative emotions and loneliness

The behavioural studies included in this systematic review consistently showed a significant positive correlation between loneliness and general negative emotions such as depression, anxiety, and stress. These results are consistent with the emerging body of evidence indicating a critical role of loneliness in precipitating depression, anxiety, and stress (Varma *et al*., 2021; Wang *et al*., 2022). Most existing research supported a bidirectional relationship between loneliness and negative emotions. Specifically, loneliness could make individuals more susceptible to experiencing negative emotions (Heinrich and Gullone, 2006; Erzen and Çikrikci 2018), and elevated levels of negative emotions can result in a lack of interest in social interactions and emotional fatigue, thereby increasing the likelihood of feeling lonely (Heinrich and Gullone, 2006; Wolters *et al*., 2023). The very limited neuroimaging evidence also indicates loneliness may be associated with increases in functional level of the brain negative emotion system, specifically the insula and amygdala. However, further studies need to provide more conclusive evidence supporting this association.

However, similar to the studies on positive emotions, existing research had mostly focused on the relationship between loneliness and general negative emotions, while evidence is lacking on whether loneliness is associated with changes in negative social emotions specifically. Although general negative emotions such as fear and anger are related to negative feelings towards social contexts, it has been shown that these types of negative emotions can dissociate (Cândea and Szentagotai-Tătar, 2018). Also, while general negative emotion processing was previously found to be performed by brain circuitries including the amygdala, anterior cingulate cortex and insula, negative social emotions may additionally implicate regions of the brain social network such as the DMPFC, superior temporal gyrus and the temporoparietal junction (Lam *et al*., 2021). Therefore, future studies should specifically examine the relationship between loneliness and negative social emotions.

Another issue was that existing studies tended to rely on questionnaire assessments rather than using task paradigms to assess negative emotions. While questionnaires focus on assessing an individual's subjective negative feelings and emotions, task paradigms tend to measure an individual's behavioural tendencies such as sensitivity and avoidance to negative stimuli. A comprehensive evaluation of negative emotions should encompass both subjective experiences and behavioural tendencies, with the latter being assessed using task paradigms.

### The relationship between emotion regulation system and loneliness

We reviewed 3 studies (Wong *et al*., 2019b; Shao *et al*., 2020; Lieberz *et al*., 2022) which assessed the relationship between loneliness and the brain emotion regulation circuitry in relation to negative emotion regulation. These findings indirectly supported an association between loneliness and negative emotions. However, other studies did not determine whether the emotion regulation circuitry pertained to positive or negative emotion regulation, making the interpretation of their findings ambiguous. The emotional regulation system associated with loneliness particularly concerned the prefrontal cortex. For instance, evidence indicated a negative correlation between loneliness and the activation of the ventrolateral prefrontal cortex (VLPFC) specifically during watching negative social stimuli (Cacioppo *et al*., 2009). Additionally, loneliness was found to be associated with reduced resting-state functional connectivity of the VLPFC and other cortical networks (Tian *et al*., 2017; Dong *et al*., 2021). However, evidence from structural imaging studies revealed that in healthy individuals, higher levels of loneliness were associated with increased gray matter volumes in the DLPFC. This structural volume increase could be a consequence of compensatory responses to reduced functioning of the LPFC, as observed among individuals with mood dysfunctions (Liu *et al*., 2016). This speculation needs to be tested using longitudinal design with both structural and functional imaging analyses.

### The relationship between emotion regulation system and loneliness

Given loneliness originates from insufficient social relations, it is surprising that only one neuroimaging study revealed reduced functional activity of the brain social cognition system, specifically in the DMPFC, among lonely individuals while processing pleasant social versus pleasant nonsocial stimuli (Cacioppo *et al*., 2009). The DMPFC is implicated in both self-referential and social processing (Meyer and Lieberman, 2018). Indeed, it was proposed that the self-referential function of the DMPFC might form the basis for this region to support social cognition and emotions, as one understands the mind of others through self-reflection (Yeshurun *et al*., 2021). It has also been shown that the DMPFC is specifically involved in theory of mind processes based on appraising social interactions (Wagner *et al*., 2016). Interestingly, the social stimuli used in Cacioppo *et al*. (2009) also extensively portrayed social interactions. Thus, the finding suggested that the reduced DMPFC activity may account for lower capacity to derive social pleasure through empathizing with others in lonely individuals. Clearly, more research is needed to replicate the DMPFC activity decrease related to loneliness, and to examine potential alterations in other regions implicated in social cognition, such as the temporoparietal junction.

### Other neuroimaging findings related to loneliness

Besides those reviewed here, there exist several recent neuroimaging studies which were not included in the present review due to non-focus on positive or negative emotions (Spreng *et al*., 2020), being a review rather than original investigation (Lam *et al*., 2021), focus on social position rather than loneliness per se (Baek *et al*., 2022), or being published after our search end date (Baek *et al*., 2023). Nevertheless, these studies reported relevant findings that are consistent with those presented here. Spreng *et al*. (2020) revealed that loneliness measured by a single question was most prominently associated with altered resting-state functional connectivity, white-matter tract and grey matter volumes in the DMN, including key regions such as the DMPFC. Lam *et al*. (2021) systematically reviewed the neuroimaging literature of loneliness, and concluded that loneliness was associated with functional and structural alterations in the medial and lateral PFC, insula, amygdala, hippocampus, and ventral striatum. Baek *et al*. (2022) reported that individuals who occupy more central positions in the social network exhibited greater neural similarity to their peers in the DMN such as the DMPFC, while Baek *et al*. (2023) further showed that lonely individuals displayed neural dissimilarity to peers within similar DMN regions. Given the DMN is considered as a common neural substrate underlying both self- and other-processing (Yeshurun *et al*., 2021), more research is critically needed that investigates the association of loneliness and affective processing in the brain across intra- and inter-individual contexts.

### The bivalence emotion model of loneliness: limitations and future directions

On the basis of the evidence reviewed, we propose a bivalence emotion model of loneliness, which posits that changes in both positive and negative emotion systems play significant roles in precipitating loneliness. The existing findings generally indicate that loneliness is negatively associated with positive emotions, but positively associated with negative emotions. This pattern was observed across studies that measured emotions using questionnaires, and those that examined brain circuitries involved in emotion processing and regulation. However, several critical gaps existed regarding this model. First, evidence for the negative relationship between loneliness and positive emotions needs to be established among adolescents and middle-aged adults. Second, further research needs to test whether loneliness is associated with positive and negative emotions in general, or positive and negative social emotions in particular, by employing both questionnaires and paradigm measures of emotions. Third, more research needs to be conducted on the relationship between loneliness and emotion regulation functions measured using questionnaires or behavioural paradigms. Importantly, the regulation of positive and negative emotions needs to be differentiated. Fourth, future neuroimaging research should simultaneously examine structural and functional data, as well as their relationships, to elucidate whether structural increase could be a compensatory response to functional impairment in lonely individuals. Neuroimaging research should also simultaneously assess behavioural/questionnaire measures of emotions and neural patterns, as well as their associations, to strengthen inferences about psychological meanings of the neural findings. Finally, longitudinal studies are urgently needed to establish the causal relationship between positive and negative emotion functions and loneliness, as well as identifying dynamic changes in the brain during the trajectory of loneliness development.

## Conclusion

Existing evidence provides preliminary support for a bivalence model of loneliness, which posits loneliness is related to increase of negative emotions and decrease of positive emotions. Notwithstanding, several critical limitations existed that may be addressed by future studies by including adolescent and middle-aged participants, measuring both general and social emotions using questionnaires and task paradigms, assessing and differentiating between positive and negative emotion regulation functions, examining the relationship between brain structural and functional alterations in lonely individuals, and carrying out longitudinal analysis.

## Supplementary Material

kkad029_Supplemental_File

## Data Availability

Data will be made available on request.
